# Impact of the COVID-19 pandemic on emergency admission for patients with stroke: a time series study in Japan

**DOI:** 10.1186/s42466-021-00163-8

**Published:** 2021-12-13

**Authors:** Takuaki Tani, Shinobu Imai, Kiyohide Fushimi

**Affiliations:** 1 Tokyo Medical and Dental University Graduate School of Medical and Dental Sciences, 1-5-45 Yushima, Bunkyo-ku, Tokyo, 113-8510 Japan; 2 Clinical Research Center National Hospital Organization, 2-21 Higashigaoka, Meguro-ku, Tokyo, 152-8621 Japan; 3 Tokyo University of Pharmacy and Life Sciences, 1432-1 Horinouchi, Hachiozi-shi, Tokyo, 192-0392 Japan

**Keywords:** COVID-19, Stroke, Emergency admission, Time series

## Abstract

**Background:**

Appropriate treatment of stroke immediately after its onset contributes to the improved chances, while delay in hospitalisation affects stroke severity and fatality. This study aimed to determine the impact of the coronavirus disease 2019 (COVID-19) pandemic on emergency hospitalisation of patients with stroke in Japan.

**Methods:**

This was an observational study that used nationwide administrative data of hospitalised patients diagnosed with stroke. We cross-sectionally observed patients’ background factors during April and May 2020, when the COVID-19 pandemic-related state of emergency was declared; we also observed these factors in the same period in 2019. We also modelled monthly trends in emergency stroke admissions, stroke admissions at each level of the Japan Coma Scale (JCS), fatalities within 24 h, stroke care unit use, intravenous thrombolysis administration, and mechanical thrombectomy implementation using interrupted time series (ITS) regression.

**Results:**

There was no difference in patients’ pre-hospital baseline characteristics between the pre-pandemic and pandemic periods. However, ITS regression revealed a significant change in the number of emergency stroke admissions after the beginning of the pandemic (slope: risk ratio [RR] = 0.97, 95% confidence interval [CI]: 0.95–0.99, *P* = 0.027). There was a significant difference in the JCS score for impaired consciousness in emergency stroke, which was more severe during the pandemic than the pre-pandemic (JCS3 in level: RR = 1.75, 95% CI: 1.29–2.33, *P* < 0.001). There was no change in the total number of fatalities with COVID-19, compared with those without COVID-19, but there were significantly more fatalities within 24 h of admission (fatalities within 24 h: RR = 1.75, 95% CI: 1.29–2.33, *P* < 0.001).

**Conclusions:**

The infection prevalence of COVID-19 increased the number of fatalities within 24 h as well as the severity of illness in Japan. However, there was no difference in baseline characteristics, intravenous thrombolysis administration, and mechanical thrombectomy implementation during the COVID-19 pandemic. A decrease in the number of patients and fatalities was observed from the time the state of emergency was declared until August, the period of this study.

## Background

The coronavirus 2019 (COVID-19) pandemic has been characterised by rapid spread of infection, necessitating the implementation of various preventive measures worldwide [1]. With various countermeasures in place against the COVID-19 infection, hospitalisation rates have decreased in overseas emergency hospitals, and a similar trend has been reported across urban areas in Japan [2–4].

Stroke is the second most common cause of death worldwide, the fourth most common cause of death in Japan, and a common cause of emergency hospitalisation.

The most common neurological manifestations in COVID-19-infected patients are cerebral infarction and mortality. Therefore, the prevalence of COVID-19 infection may have an impact on the severity of stroke in patients [5, 6].

Healthcare delivery for stroke can be considered to be strongly influenced by COVID-19 infection [7]. Appropriate treatment, such as administration of intravenous thrombolysis (IVT) and implementation of mechanical thrombectomy (MT), immediately after the onset of stroke can contribute to the improved chances of survival; however, the time of access to the hospital from the onset of stroke is critical [8–11]. Therefore, since the delay in hospitalisation from the onset of stroke may affect severity and fatality, it is important to understand the emergency hospitalisation status of patients with stroke due to COVID-19 infection.

The Japanese government has implemented various strategies to prevent the spread of COVID-19, among which was the declaration of a state of emergency from 7 April to 25 May 2020. The public was requested to take care to prevent the spread of infection at medical institutions or when travelling, and the government instructed people to contact medical institutions before seeing a doctor if they had fever or other symptoms of COVID-19 [12].

Previous studies focusing on COVID-19 have shown a highly significant decrease in the number of visits to emergency cardiology departments during lockdowns [2, 3]. The fear of a pandemic may have led to changes in patient behaviour, such as avoiding hospitalisation, even when symptoms are present [13]. According to Japanese regional ambulance activation data, the proportion of emergency calls in the first half of 2020 was lower than that during the same period in 2019 [14]. It is possible that the COVID-19 infection has reduced the rate of access to medical care for patients with stroke in Japan [15]. One previous study aimed to clarify the effects of the COVID-19 pandemic in a comprehensive stroke centre in Japan; the results showed that true emergencies appear to be associated with fewer emergency hospital visits and hospitalisations for stroke [15].

In this context, the purpose of this study was to determine the impact of the COVID-19 pandemic on the admission of patients with stroke to emergency hospitals and the resulting changes in stroke severity and fatality throughout Japan.

## Methods

### Study design

This observational study used nationwide administrative data from patients with stroke who had been admitted to emergency departments between April 2018 and August 2020. Interrupted time series (ITS) regression analysis was conducted; the interrupted time was from April to May 2020, when the state of emergency was declared.

### Patient selection

We extracted data from inpatients who were diagnosed with cerebral infarction at the time of admission from April 2018 to August 2020. The International Classification of Diseases, 10th Revision (ICD‐10) codes were used for the identification of cerebral infarction (I63). Patients who visited the emergency room were included in our study, as well as patients who visited an emergency hospital and were hospitalized.

### Data source and variables

All data were obtained from the Diagnosis Procedure Combination, which is a national database of acute care inpatients in Japan. Details of the database are presented in the cited literature [16–19].

The database encompasses hospital ID, patient ID, age, sex, weight and height, diseases, comorbidities according to ICD-10 codes, length of stay (LOS), Barthel Index, Modified Rankin Scale (mRS) at discharge, admission and discharge destination, Japan Coma Scale (JCS) at admission, onset days (four categories: ≤ 3 days, 4–7 days, ≥ 8 days, asymptomatic), and ambulance use.

We divided patients into the following six groups as the probability of lifestyle-related diseases increases from approximately 40 years of age: 0–44, 45–54, 55–64, 65–74, 75–84, and ≥ 85 years [20]. Body mass index at admission was categorised based on the modified World Health Organization classification: < 18.4 kg/m^2^_,_ 18.5–24.9 kg/m^2^, 25.0–29.9 kg/m^2^, 30.0–34.9 kg/m^2^, > 35.0 kg/m^2^, and missing values [21].

The Charlson Comorbidity Index (CCI) was calculated using Quan’s protocol [22, 23], with patients’ comorbidity conditions classified into 17 categories. Each condition had a weighted score of 1, 2, 3, or 6 depending on the mortality risk associated with the case. Individual scores were summed up to obtain the total CCI scores. We divided the total CCI scores into the following four groups: 0, 1, 2, and ≥ 3. The JCS is a unique Japanese method of assessing the level of consciousness, similar to the Glasgow Coma Scale. A JCS code of 0 denotes a lack of consciousness, while a code of 1–3 represents being awake without stimuli. A code of 10–30 signifies the ability to be roused by some stimuli, and 100–300 signifies the state of not being roused [24]. We divided the JCS into four groups according to the number of digits: 0, 1, 2, and 3. We extracted the data as follows: using stroke care unit (SCU), IVT administration, and MT implementation.

To study the impact of COVID-19 infection, we considered the pandemic period to be from 1 April to 31 May 2020 even though the state of emergency was from 7 April to 25 May 2020 because of the reliance on monthly hospitalisation data. The average number of new infections per day in Japan was 475.7 between 1 April and 31 May, and 505.7 between 7 April and 25 May [1]. It should be noted that the data used in this study are hospitalisation data, not epidemiological data on stroke [25].

The requirement for informed consent was waived because of the use of anonymised data. Study approval was obtained from the Institutional Review Board of Tokyo Medical and Dental University.

### Statistical methods

Continuous variables were expressed as mean and standard deviation and categorical variables as the number of subjects and percentages. The chi-square test and t-test were used to analyse differences in the characteristics of patients with stroke between April and May 2019, as well as April and May 2020, for discrete and continuous variables. Because of the large number of subjects in this study, the standardised mean difference (SMD), which is unaffected by the number of subjects, was calculated, and the significant difference in the test was examined. The baseline characteristics were compared with an absolute standardised difference of < 10% considered to denote negligible imbalances between the pandemic and pre-pandemic periods [26].

We used ITS regression to model monthly trends in the number of emergency stroke admissions, number of stroke admissions at each level of the JCS (0, 1, 2, and 3), number of fatalities within 24 h or otherwise, SCU admission, administration of IVT, and implementation of MT [27–29]. The interrupted time was April to May 2020, when the state of emergency was declared.

The Poisson distribution was used as the link function for all models, with the months of April and May 2020 being points in the equation to verify the difference in the impact of the declaration of the state of emergency. All months from the declaration of the state of emergency were entered into the ITS as trend points in order to see the impact from the declaration of the state of emergency onwards. In the ITS equation excluding hospital admissions, the number of hospital admissions for that point was log transformed and put in as an offset term to adjust for the effect of the overall number.

We hypothesised a level change model considering the slope change. All outcome variables used in the ITS regression were examined for a unit roof using the Dickey–Fuller test [30]. We included fixed effect terms for the year and trend effect. We adjusted the seasonal trend if there was autocorrelation in the model. After checking and adjusting the model for seasonality and trends, autocorrelation and partial autocorrelation were plotted to check for autocorrelation of residuals. All tests were two-tailed, and the threshold for significance was set at *P* < 0.05. Statistical analyses were performed using R statistical software (version 3.3.2; R Foundation for Statistical Computing, Vienna, Austria).

## Results

**Fig. 1:**
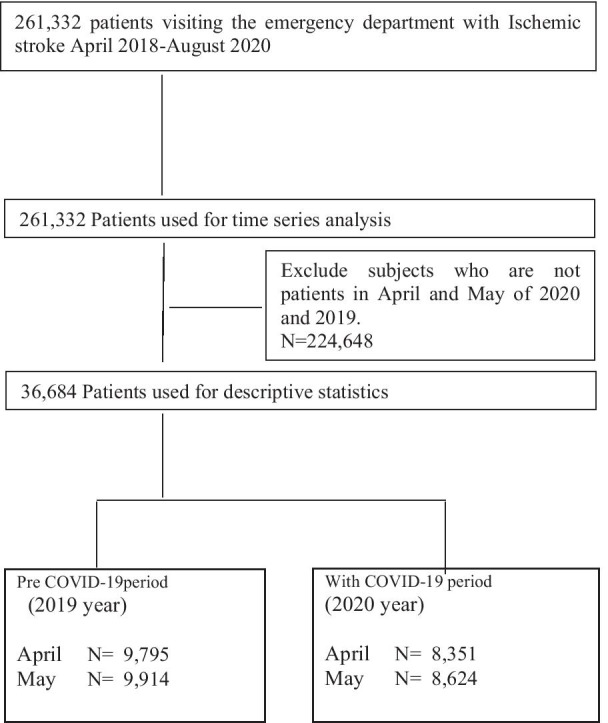
Flowchart of the study cohort

Figure 1 shows that a total of 261,332 eligible patients were identified between April 2018 and August 2020. During the state of emergency, there were 8,351 admitted patients in April, and 8,624 in May. In 2019, 9,795 and 9,914 patients were admitted in April and May, respectively.

Table 1 shows the preadmission baseline characteristics of patients admitted in April 2019 and May 2020. There was no difference in any of the preadmission baseline variables, age, gender, mRS, or CCI between the pandemic and pre-pandemic periods.

**Table 1:** Baseline characteristics of patients with stroke

Variables	Pre-COVID-19 period	COVID-19 period	SMD	*P* value
n	19,709	16,975		
Age, N, %			0.021	0.649
0–44	308 (1.6)	242 (1.5)		
45–54	916 (4.6)	752 (4.4)		
55–64	1790 (9.1)	1508 (8.9)		
65–74	4693 (23.8)	3986 (23.5)		
75–84	6702 (34.0)	5846 (34.4)		
85-	5300 (26.9)	4641 (27.3)		
Sex (Female), N, %	8395 (42.6)	7234 (42.6)	< 0.001	0.976
BMI, N, %			0.046	0.002
< 18.5	2264 (11.7)	1964 (11.7)		
18.5–24.9	11,111 (57.2)	9521 (56.8)		
25–29	3704 (19.1)	3403 (20.3)		
30–34	679 (3.5)	547 (3.3)		
35 and over	693 (3.6)	600 (3.6)		
NA	970 (5.0)	714 (4.3)		
mRS at admission, N, %			0.028	0.324
0	8658 (44.1)	7458 (44.0)		
1	3650 (18.6)	3073 (18.1)		
2	2451 (12.5)	2186 (12.9)		
3	1896 (9.6)	1704 (10.1)		
4	1936 (9.9)	1674 (9.9)		
5	842 (4.3)	663 (3.9)		
Other	221 (1.1)	185 (1.1)		
CCI, N, %			0.014	0.618
0	16,717 (84.8)	14,362 (84.6)		
1	2538 (12.9)	2191 (12.9)		
2	399 (2.0)	377 (2.2)		
3	55 (0.3)	45 (0.3)		
Admission pathway, N, %			0.049	0.002
Home	16,315 (82.8)	14,103 (83.1)		
Facility	1456 (7.4)	1323 (7.8)		
Transfer	1917 (9.7)	1524 (9.0)		
Else	21 (0.1)	25 (0.1)		
Stroke of onset time, N, %			0.052	< 0.001
3 days or less	16,629 (84.6)	14,438 (85.2)		
4–7 days	928 (4.7)	861 (5.1)		
8 days or more	1796 (9.1)	1471 (8.7)		
Asymptomatic	302 (1.5)	172 (1.0)		
Ambulance, N, %	9950 (50.5)	8853 (52.2)	0.033	0.002

*BMI* body mass index, *JCS* Japan Coma Scale, *BI* Barthel Index, *CCI* Carlson Comorbidity Index, *SMD* standardised mean difference

In BMI, admission pathway, onset category of stroke, and use of ambulance, there was a significant difference in the pandemic and pre-pandemic periods, but the effect size was not significant.

Table 2 shows the impact of the pandemic period on healthcare utilisation and severity of stroke in emergency hospitalisations.

**Table 2:** Mean JCS score, LOS, fatalities, fatalities in 24 h, mRS, and discharge during the COVID-19 pandemic period

Variables	Pre-COVID-19 period		COVID-19 period		Diff	SMD	*P* value
SCU	2433	(12.3)	2153	(12.7)	0.4	0.01	0.336
IVT, N, %	1125	(5.7)	1096	(6.5)	0.8	0.031	0.003
MT, N, %	868	(4.4)	807	(4.8)	0.4	0.017	0.115
JCS at admission, N, %						0.071	< 0.001
0	9813	(49.8)	7896	(46.5)	− 0.33		
1	7540	(38.3)	6897	(40.6)	2.3		
2	1621	(8.2)	1425	(8.4)	0.2		
3	735	(3.7)	757	(4.5)	0.8		
LOS, Mean, SD	31.32	(34.59)	28.01	(27.0)	− 3.31	0.107	< 0.001
Fatalities, N, %	1070	(5.4)	878	(5.2)	− 0.2	0.011	0.285
Fatalities in 24 h, N, %	37	(0.2)	66	(0.4)	0.2	0.038	< 0.001
mRS at discharge, N, %						0.041	0.03
0	1970	(10.0)	1559	(9.2)	− 0.8		
1	4310	(21.9)	3679	(21.7)	− 0.2		
2	3493	(17.8)	3111	(18.4)	0.6		
3	2720	(13.8)	2294	(13.6)	− 0.2		
4	3886	(19.8)	3381	(20.0)	0.2		
5	2163	(11.0)	1997	(11.8)	0.8		
Other	1100	(5.6)	901	(5.3)	− 0.3		
Discharge destination, N, %						0.038	0.004
Home	10,666	(54.1)	9050	(53.3)	− 0.8		
Facility	1706	(8.7)	1375	(8.1)	− 0.6		
Hospital	6252	(31.7)	5661	(33.3)	1.6		
Other	1085	(5.5)	889	(5.2)	− 0.3		

*SCU* Stroke care unit, *IVT* intravenous thrombolysis, *MT* Mechanical thrombectomy, *LOS* length of hospital stay, *JCS* Japan Coma Scale, *mRS* modified Ranking Scale

SCU and MT were not significantly different under the COVID-19 emergency declaration; for IVT, there was a significant difference [pandemic (IVT): 1,096(6.5%) vs pre-pandemic (IVT): 1,125(5.7%), diff = 0.8, SMD = 0.031, *P* < 0.001].

There was a significant difference in the JCS disorientation score between the pandemic and pre-pandemic periods: more patients were hospitalised with severe disorientation during the pandemic [pandemic (JCS0, JCS1, JCS2, JCS3): 7,896 (46.5%), 6,897 (40.6%), 1,425 (8.4%), and 757 (4.5%) vs pre-pandemic (JCS0, JCS1, JCS2, JCS3): 9,813 (49.8%), 7,540 (38.3%), 1,621 (8.2%), and 735 (3.7%), respectively; SMD = 0.071, *P* < 0.001].

There was no significant difference in fatality in the pandemic and pre-pandemic periods, but there was a significant difference in 24-h fatality upon admission [pandemic: 37 (0.2) vs. pre-pandemic: 66 (0.4), SMD = 0.038, *P* < 0.001]. There was also a significant difference in the category of discharge destination, with more patients being transferred to other hospitals [pandemic (home, facility, hospital, others): 9,050, 1,375, 5,661, and 889, respectively vs pre-pandemic (home, facility, hospital, others): 10,666, 1,706, 6,252, and 1085, respectively; SMD = 0.038, *P* < 0.004].

Table 3 shows the results of the ITS regression for the number of patients with stroke from April 2018 to August 2020. There was a significant change in the slope of the number of emergency stroke admissions assessed by ITS regression between t the pandemic and pre-pandemic periods (slope: risk ratio [RR] = 0.97, 95% confidence interval[CI]: 0.95–0.99, *P* = 0.027).

**Table 3:** Interrupted time series analysis for the number of patients with stroke from April 2018″ to 31 August 2020

	Change in level			Change in slope		
	RR	95% CI	*P* value	RR	95% CI	*P* value
Hospital admissions	0.97	(0.90, 1.06)	0.5	0.97	(0.95, 0.99)	0.027
JCS0	0.95	(0.92, 0.98)	< 0.001***	1.00	(0.99, 1.00)	0.3
JCS1	1.04	(1.01, 1.07)	0.023*	1.01	(1.00, 1.02)	0.3
JCS2	1.02	(0.96, 1.09)	0.5	0.99	(0.97, 1.01)	0.5
JCS3	1.24	(1.14, 1.36)	< 0.001***	1.01	(0.99, 1.04)	0.3
Fatalities within 24 h	1.75	(1.29, 2.33)	< 0.001***	1.15	(1.04, 1.27)	0.004**
Fatalities	0.96	(0.80, 1.13)	0.6	0.93	(0.88, 0.98)	0.017*
SCU	1.00	(0.95, 1.05)	0.9	1.06	(1.04, 1.08)	< 0.001***
IVT	0.96	(0.88, 1.05)	0.4	1.00	(0.97, 1.03)	0.9
MT, N, %	1.02	(0.94, 0.95)	0.5	1.00	(0.98, 1.02)	0.9

*RR* risk ratio, *CI* confidence interval, *JCS* Japan Coma Scale, *SCU* Stroke care unit, *IVT* intravenous thrombolysis, *MT* Mechanical thrombectomy

***P < 0.001, **P < 0.01, *P < 0.05

There was a significant difference in JCS levels 0, 1, and 3, and impaired consciousness in emergency stroke was more severe during the pandemic period than in the pre-pandemic (JCS level 3: RR = 1.75, 95% CI: 1.29–2.33, *P* < 0.001) period. During the pandemic period, the number of fatalities within 24 h of admission was significantly higher than the number of fatalities more than 24 h after admission. However, there was no change in total number of fatalities compared with the pre-pandemic period (Fatalities within 24 h: RR = 1.75, 95% CI: 1.29–2.33, *P* < 0.001; Fatalities: RR = 0.96, 95% CI: 0.80–1.13, *P* < 0.6). After the onset of the pandemic, although the number of fatalities was significantly higher within 24 h of hospitalisation, the overall number of fatalities was slightly decreased compared with the pre-pandemic period (slope: fatalities within 24 h: RR = 1.15, 95% CI: 1.04–1.27, *P* = 0.004; fatalities: RR = 0.93, 95% CI: 0.88–0.98, *P* < 0.017).

There was no significant effect on IVT, MT, and SCU during the pandemic. However, SCU was slightly increased after the onset of the pandemic (slope of SCU: RR = 1.06, 95% CI: 1.04–1.08, *P* < 0.001).

Although no unit root was found for all the variables used in the ITS regression, no autocorrelation or partial autocorrelation was observed in the adjusted model.

**Fig. 2:**
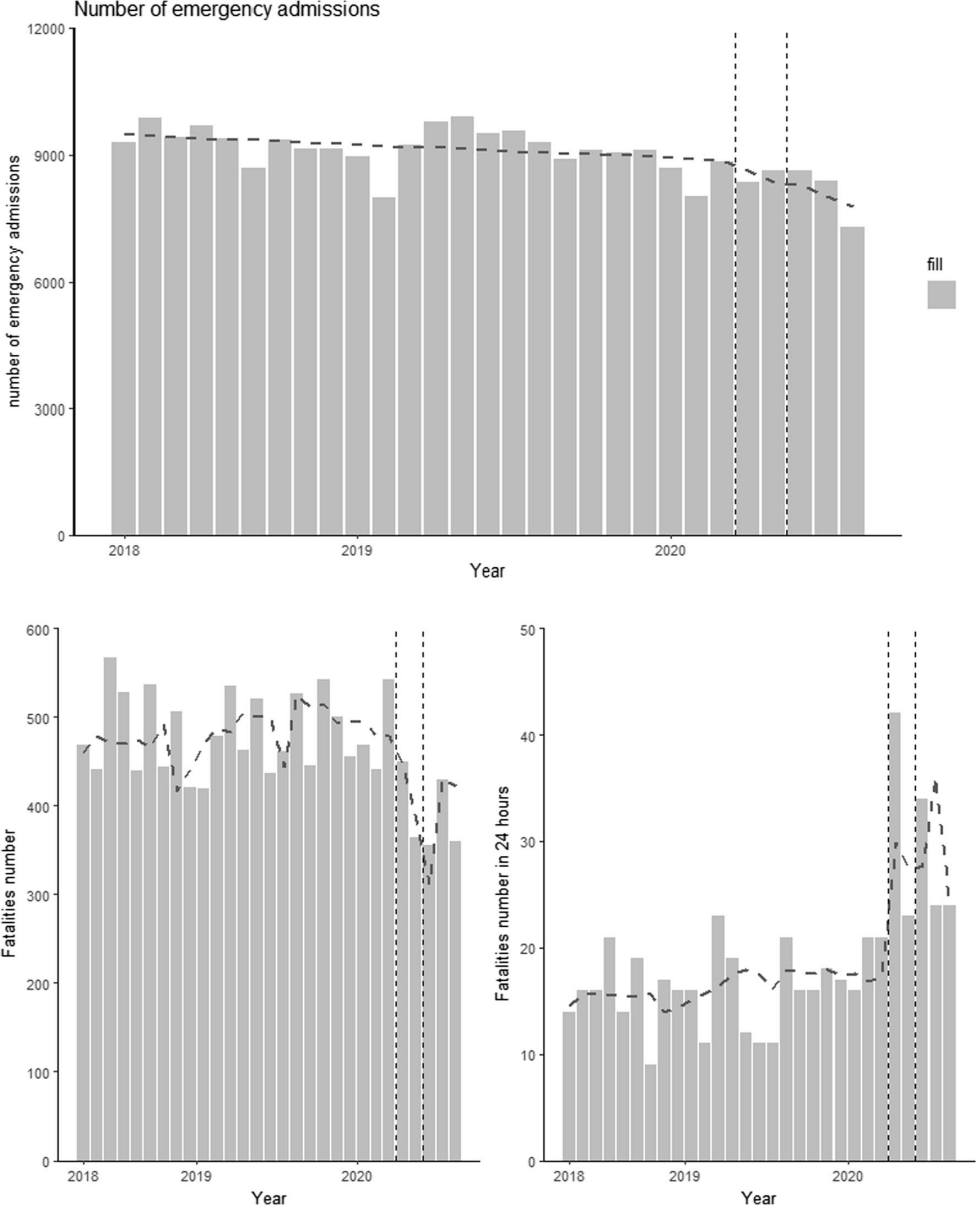
Interrupted time series analysis of emergency stroke admissions in the COVID-19 pandemic

Figure 2 shows the plot of ITS for the number of patients admitted, f total number of fatalities, and fatalities within 24 h with predicted numbers for ITS regression.

## Discussion

In this study, we revealed that stroke severity and the number of fatalities within 24 h of admission increased. However, there was no change in the treatment (IVT and MT), total number of fatalities, and patient characteristics, such as age, sex, and dependency. This suggests that under the declared state of emergency during the COVID-19 pandemic, there was no problem in the provision of medical care for stroke, from emergency transport to treatment. On the other hand, after the declaration of the state of emergency, the number of patient admissions and post-hospitalization fatalities showed a downward trend, indicating an increase in the number of patients who were not admitted.

In this study, the authors evaluated the changes in the severity of stroke emergencies during the COVID-19 pandemic in Japan. We found that the number of fatalities within 24 h of admission and the number of patients with a high level of consciousness at admission increased during the COVID-19 pandemic compared to the pre-pandemic period. However, the overall fatality rate was not affected, suggesting that a group of patients with originally-fatal severity may have died within 24 h as the severity of the disease increased [31].

We found no difference in the rates of IVT and MT compared with the pre-pandemic period. Since IVT and MT have a time limit from the onset of treatment, the change in these rates was an indicator of the change in transport status from the onset of the treatment to reaching the hospital [32]. In a German study that compared the implementation rates of IVT and MT during the COVID-19 pandemic, there was no change in the implementation of the former but a decrease in the implementation rate of the latter [5]. In Japan, as the number of infected people was about 1/20th the number in Germany at the time of the study, it was believed that the pandemic did not have an effect on transport status; however, if COVID-19 spreads, it could have an impact on transport.

The number of emergency hospital admissions of stroke patients during the pandemic period did not change compared to the pre-pandemic period. However, hospital admissions showed a decreasing trend in the number of patients from the pandemic hroughout the month of August in the observation period. A cross-sectional comparison between the results for 2020 and 2019 during the pandemic gives the impression of a decrease, but the number of patients in 2019 increased when looking at the time series. Further, there was no difference in the level of change when looking at the ITS results. However, a difference in the ITS slope was observed, and the number of patients had been decreasing in August, since the pandemic. These results were not consistent with those of a previous study conducted in Japan.

The previous study aimed to determine the impact of the COVID-19 pandemic in a comprehensive urban stroke centre. The results showed that the number of emergencies caused by COVID-19 showed a decreasing trend [15]. There were two major possible reasons for this. First, the previous studies were conducted at a single institution and in an area with a relatively high number of COVID-19 infections, which might have been strongly affected by the COVID-19 epidemic. Because this study used data that encompassed about half of the total population of Japan and looked at the impact after the declaration of the state of emergency, when COVID-19 infections were increasing throughout Japan, there might have been changes in the COVID-19 impact in different regions of Japan [33].The second reason was that the previous study had a short observation period of a few months and thus did not take into account such factors as yearly variations. In the present study, the period used was approximately two years of data from April 2018 to August 2020, and the analysis was adjusted to account for yearly variations. It was known that the number of stroke patients fluctuates seasonally, and therefore, it was necessary to consider long-term variations, which led to different results between this study and previous studies [34].

This study revealed a decreasing trend in the number of patients hospitalized for cerebral infarction from the time after the declaration of the state of emergency until August, the month studied in this paper, although we could not confirm a decrease in the number of patients hospitalized for cerebral infarction under the declaration of the state of emergency. This may have been due to the fact that the number of infected people in Japan had been on an increasing trend since late July 2020, exceeding the maximum number under the declaration of the state of emergency [1].

The overall number of fatalities and new patients did not decrease under the declaration of a state of emergency based on the results of the ITS analysis but trended downward after the declaration of a state of emergency. A previous study examining the impact of the COVID-19 pandemic on access to emergency hospitals found an increase in the number of critically-ill hospitalized patients and a decrease in the number of new patients. The aforementioned study found an increase in the number of critically ill patients in hospitals and a decrease in the number of new patients. The aforementioned studies indicated that the number of hospitalized patients decreased because patients avoided hospitalization for fear of COVID-19 infection [3, 5]. This study revealed that the number of patients and associated fatalities in Japan has been on a downward trend since the declaration of the state of emergency. This suggests that hospitalized patients with high severity of illness may not have been hospitalized because they died at home.

Despite the importance of the findings, this study has certain limitations. In this study, we looked at changes in outcomes in a cross-sectional observation comparing April and May of 2020 with April and May of 2019 under a declared state of emergency and a time series from 2018 to 2020. In the cross-sectional comparison, there was no change in effect size even though the difference in JCS and 24-h mortality was significant with little impact on outcomes under the declaration of emergency. However, the results of the time series analysis were combined with the cross-sectional analysis to assess the impact of COVID-19. The data are related to hospitalisation and are not epidemiological data on stroke. Therefore, this study does not capture changes in patients with stroke who were not hospitalised. Second, while the state of emergency was from 7 April to 25 May, the pandemic period in this study was from 1 April to 31 May because of the reliance on monthly hospitalisation data. The average number of new infections per day in Japan was 475.7 from 1 April to 31 May and 505.7 from 7 April to 25 May; thus, the impact of the pandemic may appear to be slightly less severe in this study.

## Conclusions

The results showed that the COVID-19 pandemic increased the number of fatality and severity of hospitalization within 24 h in Japan, while a decreasing trend in the number of patients and fatalities was observed after the declaration of the emergency. However, baseline characteristics and time-dependent treatments, such as MT and IVT, were not altered. These results suggest that there were no problems with health care delivery during the COVID-19 pandemic. On the other hand, it was possible that patients who died or were mildly ill were not treated or hospitalized gradually after the declaration of the state of emergency; the persistent prevalence of COVID-19 infection may have influenced patients' behaviour to seek medical attention. During large outbreaks of infectious diseases, patients should be encouraged to seek medical attention at the onset of stroke, and such patients should be carefully monitored.

## Data Availability

The datasets supporting the conclusions of this article are available from the corresponding author upon reasonable request.

## Abbreviations

CCI: Carlson Comorbidity Index
CI: Confidence interval
IVT: Intravenous thrombolysis
JCS: Japan Coma Scale
LOS: Length of hospital stays
mRS: Modified Ranking Scale
MT: Mechanical thrombectomy
RR: Risk ratio
SCU: Stroke care unit
SMD: Standardised mean difference
IVT: Intravenous thrombolysis

## References

[1] Ministry of Health LaW, Japan. About new coronavirus infections 2020 [Available from: https://www.mhlw.go.jp/stf/covid-19/kokunainohasseijoukyou.html.

[2] Tsioufis K, Chrysohoou C, Kariori M, Leontsinis I, Dalakouras I, Papanikolaou A (2020). The mystery of "missing" visits in an emergency cardiology department, in the era of COVID-19. A time-series analysis in a tertiary Greek General Hospital. Clin Res Cardiol..

[3] Andersson C, Gerds T, Fosbol E, Phelps M, Andersen J, Lamberts M (2020). Incidence of new-onset and worsening heart failure before and after the COVID-19 epidemic lockdown in denmark: A nationwide cohort study. Circ Heart Fail..

[4] Koge J, Shiozawa M, Toyoda K (2020). Acute stroke care in the with-COVID-19 era: experience at a comprehensive stroke center in Japan. Front Neurol..

[5] Richter D, Krogias C, Eyding J, Bartig D, Grau A, Weber R (2020). Comparison of stroke care parameters in acute ischemic stroke patients with and without concurrent Covid-19. A nationwide analysis. Neurol Res Pract..

[6] Ladopoulos T, Zand R, Shahjouei S, Chang JJ, Motte J, Charles James J (2021). COVID-19: Neuroimaging features of a pandemic. Journal of Neuroimaging.

[7] Lozano R, Naghavi M, Foreman K, Lim S, Shibuya K, Aboyans V (2012). Global and regional mortality from 235 causes of death for 20 age groups in 1990 and 2010: A systematic analysis for the Global Burden of Disease Study 2010. The Lancet..

[8] Powers WJ, Rabinstein AA, Ackerson T, Adeoye OM, Bambakidis NC, Becker K (2019). Guidelines for the early management of patients with acute ischemic stroke: 2019 update to the 2018 guidelines for the early management of acute ischemic stroke: A guideline for healthcare professionals from the American Heart Association/American Stroke Association. Stroke.

[9] Neumar RW, Shuster M, Callaway CW, Gent LM, Atkins DL, Bhanji F (2015). Part 1: Executive summary: 2015 American Heart Association guidelines update for cardiopulmonary resuscitation and emergency cardiovascular care. Circulation.

[10] Hacke W, Kaste M, Bluhmki E, Brozman M, Davalos A, Guidetti D (2008). Thrombolysis with alteplase 3 to 4.5 hours after acute ischemic stroke. New Engl J Med..

[11] Davis SM, Donnan GA, Parsons MW, Levi C, Butcher KS, Peeters A (2008). Effects of alteplase beyond 3 h after stroke in the Echoplanar Imaging Thrombolytic Evaluation Trial (EPITHET): A placebo-controlled randomised trial. The Lancet Neurology..

[12] Karako K, Song P, Chen Y, Tang W (2020). Analysis of COVID-19 infection spread in Japan based on stochastic transition model. Bioscience Trends.

[13] Schull MJ, Stukel TA, Vermeulen MJ, Zwarenstein M, Alter DA, Manuel DG (2007). Effect of widespread restrictions on the use of hospital services during an outbreak of severe acute respiratory syndrome. CMAJ.

[14] Hatakeyama K, Ota J, Takahashi Y, Kawamitsu S, Seposo X (2021). Effect of the COVID-19 pandemic on heatstroke-related ambulance dispatch in the 47 prefectures of Japan. Sci Total Environ..

[15] Ota T, Shiokawa Y, Hirano T (2020). Impact of COVID-19 on stroke admissions and the medical care system in the tokyo metropolitan area. Front Neurol..

[16] Chikuda H, Yasunaga H, Horiguchi H, Takeshita K, Kawaguchi H, Matsuda S (2012). Mortality and morbidity in dialysis-dependent patients undergoing spinal surgery: Analysis of a national administrative database in Japan. Journal of Bone and Joint Surgery. American Volume.

[17] Okamura S, Kobayashi R, Sakamaki T (2005). Case-mix payment in Japanese medical care. Health Policy.

[18] Shinya-Matsuda KF, Kiyohide F (2010). Development of Casemix based evaluation system in Japan. Asian Pacific Journal of Disease Management..

[19] Miyata H, Hashimoto H, Horiguchi H, Fushimi K, Matsuda S (2010). Assessment of hospital performance with a case-mix standardized mortality model using an existing administrative database in Japan. BMC Health Services Research.

[20] Deeks A, Lombard C, Michelmore J, Teede H (2009). The effects of gender and age on health related behaviors. BMC Public Health.

[21] W. H. O. Expert Consultation (2004). Appropriate body-mass index for Asian populations and its implications for policy and intervention strategies. The Lancet.

[22] Quan H, Sundararajan V, Halfon P, Fong A, Burnand B, Luthi JC (2005). Coding algorithms for defining comorbidities in ICD-9-CM and ICD-10 administrative data. Medical Care.

[23] Charlson ME, Pompei P, Ales KL, MacKenzie CR (1987). A new method of classifying prognostic comorbidity in longitudinal studies: Development and validation. Journal of Chronic Diseases.

[24] Yumoto T, Naito H, Yorifuji T, Aokage T, Fujisaki N, Nakao A (2019). Association of Japan Coma Scale score on hospital arrival with in-hospital mortality among trauma patients. BMC Emergency Medicine.

[25] Richter D, Weber R, Eyding J, Bartig D, Misselwitz B, Grau A (2021). Acute ischemic stroke care in Germany—further progress from 2016 to 2019. Neurol Res Pract..

[26] Austin PC (2009). Balance diagnostics for comparing the distribution of baseline covariates between treatment groups in propensity-score matched samples. Statistics in Medicine.

[27] Bernal JL, Cummins S, Gasparrini A (2017). Interrupted time series regression for the evaluation of public health interventions: A tutorial. International Journal of Epidemiology.

[28] Hategeka, C., Ruton, H., Karamouzian, M., Lynd, L. D., & Law, M. R. (2020). Use of interrupted time series methods in the evaluation of health system quality improvement interventions: a methodological systematic review. *BMJ Glob Health*, *5*(10). 10.1136/bmjgh-2020-003567 PMC7559052 33055094

[29] Xiao H, Augusto O, Wagenaar BH (2021). Reflection on modern methods: A common error in the segmented regression parameterization of interrupted time-series analyses. International Journal of Epidemiology.

[30] Dabid A, Dckey WAF (1979). Distribution of the estimators for autoregressive time series with a unit root. Journal of the American Statistical Association..

[31] Chen Y, Xia F, Li Y, Li H, Ma L, Hu X (2021). Changes in characteristics, treatment and outcome in patients with hemorrhagic stroke during COVID-19. J Stroke Cerebrovasc Dis..

[32] Richter D, Eyding J, Weber R, Bartig D, Grau A, Hacke W (2021). Analysis of nationwide stroke patient care in times of COVID-19 pandemic in Germany. Stroke.

[33] Hideo Yasunaga KF, Hiroki Matsui HH, Shinya M (2013). Epidemiology and health services resarch using DPC. Asian Pacific Journal of Disease Management.

[34] Oberg AL, Ferguson JA, McIntyre LM, Horner RD (2000). Incidence of stroke and season of the year: Evidence of an association. American Journal of Epidemiology.

